# Animal tuberculosis control in a disease-free country, France: does the long and winding road really lead to eradication?

**DOI:** 10.1186/s13620-023-00258-5

**Published:** 2023-09-26

**Authors:** María Laura Boschiroli

**Affiliations:** Tuberculosis National Reference Laboratory, Animal Health Laboratory, French Agency for Food, Environmental and Occupational Health and Safety (ANSES), 94701 Maisons-Alfort, France

**Keywords:** Long-term combat, Commitment, Motivation, Innovation

## Abstract

It took France almost fifty years to attain its officially animal tuberculosis (TB) free status in 2000, granting the country a favourable position for international live animal trading. The initial TB control program has been adapted at different times in its history in order to suit changing epidemiological contexts: it was first focused on detection and elimination of infected animals while later on protecting TB free herds became a priority.

In spite of all the efforts put into the program, final eradication has still not been achieved. Instead, the eradication process has stalled, most probably due to changes in breeding practices in the last 30 years. Indeed, the beef industry has overtaken the milk industry, which has led to the occurrence of new TB risks. Novel epidemiological situations in some regions of extensive beef cattle farming, where wildlife species (wild boar, badger) are also infected, have emerged. More adapted measures have thus been implemented, progressively evaluated and improved in order to reinforce prevention of infection, to follow up with the eradication goal and to strengthen, coordinate and re-motivate field resources. These include, among others, introduction of biosecurity measures in the herd, risk based surveillance and management of wildlife and cattle, improvement of screening in the field and at the abattoir, better diagnosis, but also improvement of communication, awareness, training activities of the main field actors. Very importantly, this new plan has been established through the participation of the majority of involved stakeholders -the farmer industry, hunter associations, veterinarians, scientists and the government-, through coordinated specific steering committees and *ad hoc* working groups.

Without doubt, the main challenge for the next few years is reinforcing communication to encourage and strengthen the program in an already faltering agro-social system. In addition, it will be essential to continue sustaining national research and international collaborations to feed the program with relevant scientific data enabling the authorities to undertake the most pertinent measures for tackling the disease in the short term.

## Overview of the strategic plan for eradication of TB in France

France implemented a real collective and compulsory control campaign against animal tuberculosis (TB, formerly referred to as bovine tuberculosis (bTB)) in 1954 after an unsuccessful prophylactic phase applied in 1933, which was individual and free based. At the beginning of the fighting program, bTB affected approximately one out of four herds [1]. The economic consequences were important (reduction in the production of milk and meat, impossibility to sell living animals), but mainly undervalued livestock due to the insidiousness of the disease. The zoonotic impact of TB was also underestimated but it is considered that between 10 and 30% of human patients may have been infected by *Mycobacterium bovis* (*M. bovis*) [1]. In 1962, the Common Agriculture Policy was implemented with the objective of producing more and better food. By decree (Ministerial Decree of 14 August 1963), France implemented a control campaign based on the detection of infected animals in the herd, mainly by systematically applying the skin test periodically, but also at the abattoir by sanitary inspection of carcasses to detect TB-like lesions, in order to disclose the infection at the herd of origin. In 1964, when the “trading” Directive 64/432/EEC was adopted by the European Economic Community, the French regulations were already in harmony with it.

In the middle of the 70’s, systematic screening for TB by skin testing resulted in constraints due to the imbalance between a low proportion of herds yielding positive results despite being really infected and those which were not infected. In the 80’s, given its low positive predictive value with low disease prevalence, the simple single intradermal cervical test was complemented by the single comparative cervical test in series. Moreover, TB-infected declaration started to be done by culture confirmation.

Progressively, the control strategy included risk management at herd level, with the objective requirement to protect TB free herds by limiting the introduction of animals to those belonging exclusively to TB free herds (Ministerial Decree of 15 June 1978) that became compulsory in 1990 (loss of qualification in case of non compliance with this rule) (Ministerial Decree of 16 March 1990). The residual risk posed by herds having practiced skin testing and cull had been identified from the beginning of the 90’s. It led to the statutory decision of the systematic total slaughter of infected herds since 1999 as prevalence was low enough to financially support official compensation measures. Importantly, investigations allowing identification of the herds linked to the outbreak, either providers (upstream) or recipients (downstream), were introduced. In these epidemiologically linked herds, screening either by the skin test or by diagnostic culling and testing, greatly improved detection of new outbreaks (Ministerial Decree of 15 September 2003).

Nowadays, more than 60% of mainland regions in France are considered TB free although systematic skin control tests are no longer performed, due in part to test specificity issues resulting in excessive problems with false positive responders [2]. Other regions perform skin testing periodically (every four, three, two or every year) [2]. Such a reduction in global screening is the consequence of a favorable logical evolution of the eradication program having facilitated the disappearance of TB. However, giving up systematic screening led to a strategy adjustment, based on managing risk factors, mainly at the herd level: farmers are responsible on the one hand for the sanitary quality of introduced animals and on the other hand for biosecurity measures in order to deal with risk factors that could lead to the herd’s contamination. The quality of this sanitary supervision is controlled by a representative of the regional veterinary sanitary services.

## Evolution of breeding systems, introduction of new risk factors and reemergence of TB

**Fig. 1:**
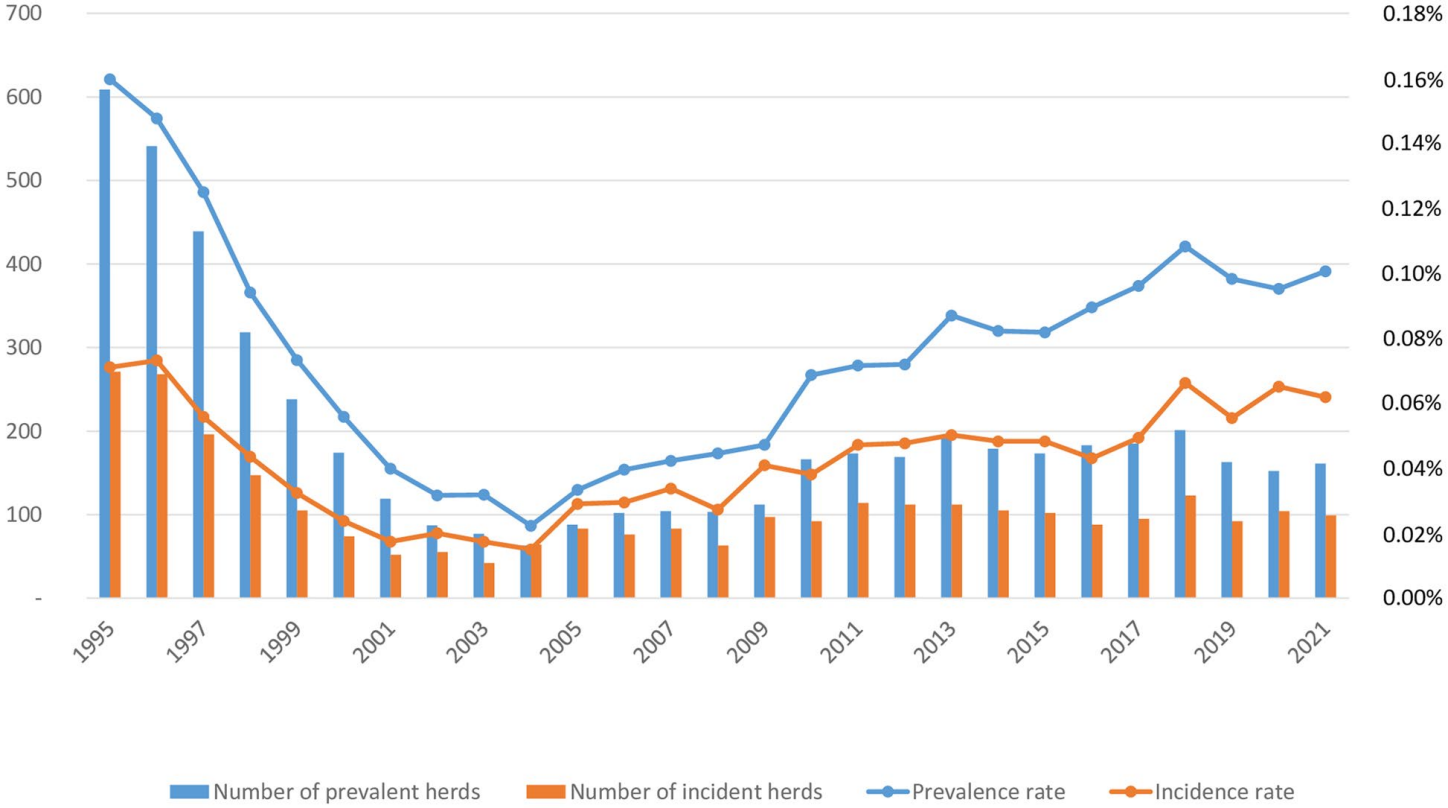
Prevalence and incidence figures of TB in France from 1995 to 2021 (Source: annual report of the Direction générale de l'alimentation  (DGAL))

**Fig. 2:**
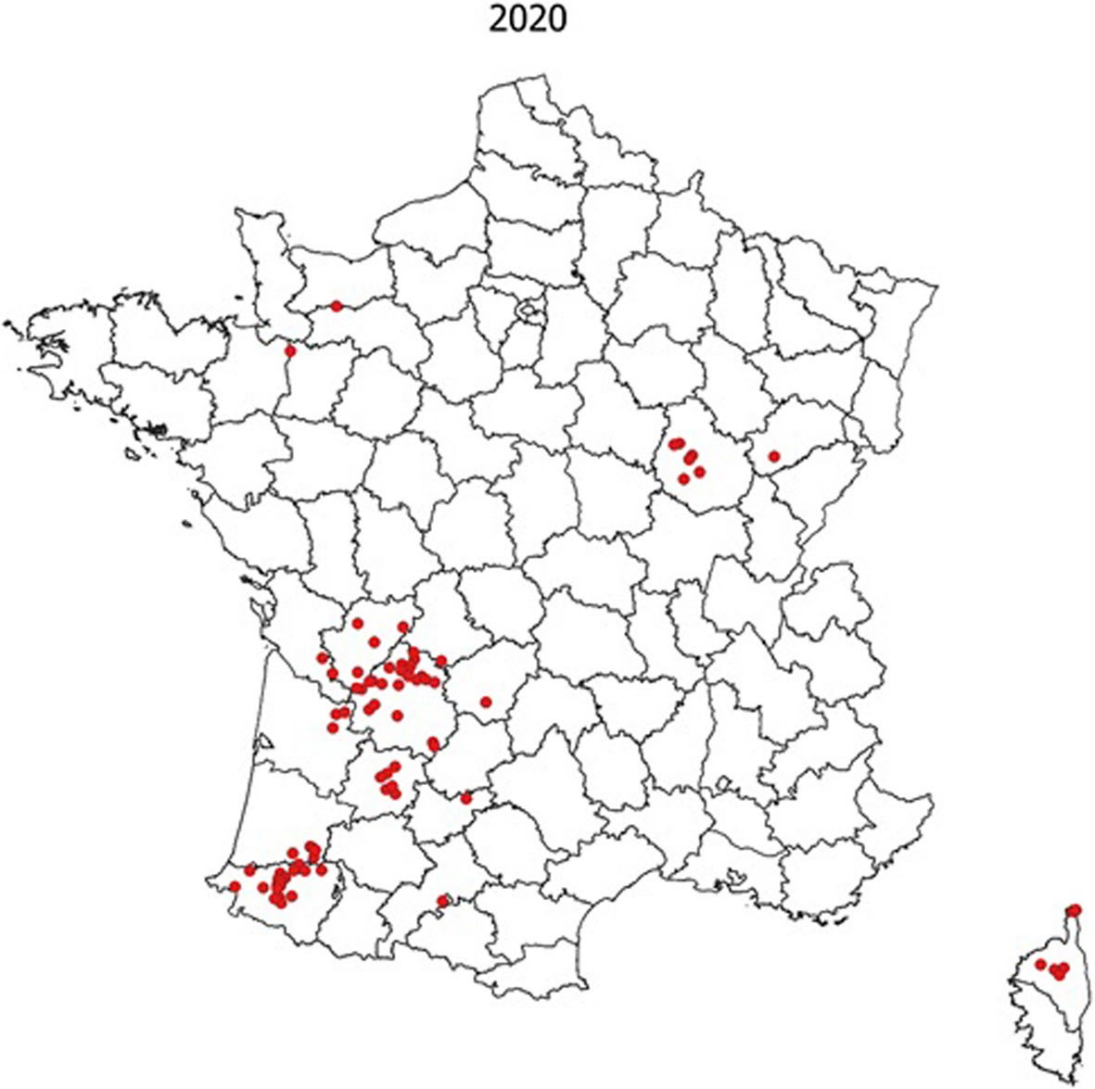
Distribution of cattle TB outbreaks in 2020. Red dots on the map represent cattle outbreaks (Source: annual report of the Direction générale de l'alimention (DGAL))

Since 2001, France has been considered OTF by the European Union (EU), with a national prevalence under 0.1%, despite a limited number of outbreaks (average 100) occurring each year (Fig. 1). The current impact of TB is essentially indirect, mainly affecting trading and hampering established markets of store cattle traded within the EU or breeder animals within the EU or to a third country. The improvement of the epidemiological profile at the start of the program has stalled since 2004 and the number of outbreaks has increased in the last years. This rise of the prevalence rate is directly linked to the particular situation in a limited number of regions where the outbreaks observed in France have been concentrated (Fig. 2).

Since 1984, when European milk quotas were imposed, and from 1992 when the EU encouraged beef cattle breeding through bonuses, the suckler herd greatly expanded. France is certainly the country where the move from dairy to suckler herds has been the most marked over the last thirty years, with a restructuring of the industry and a shift to high quality beef production leading to a very sharp drop in the number of cows in the dairy herd, being replaced partly by suckler cows from highly productive specialized breeds.

It is very difficult to make a parallel between the evolution of farm structure and the evolution of TB due to the numerous other factors suspected of playing a role. However, the analysis of the distribution and the evolution of TB in France highlights that it has had a stronger relationship with beef herds than with dairy herds in the last 15 years.

Among the risk factors linked to TB, some of them concern beef herds [3]:

- Operating a farm over several premises covering large tracts of pasture land where livestock remain for long periods which increases neighbouring risk and also increases the risk of environmental contamination mainly by interaction and contact with wildlife (badgers and wild boar),
- animals raised outdoors and living long enough to express lesions; thus, beef cows, are more likely to develop clinical signs,
- large herds are associated with reduced man power making animal surveillance more difficult,
- behavioural and restraint difficulties are more problematic in beef than in milk cattle, rendering veterinary handling more hazardous.

TB outbreaks are well limited to geographical TB-endemic areas (Fig. 2) and within each area the same *M.* *bovis* strain genotype predominates [4]. Thus, highlighting the existence of local factors allowing the propagation and maintenance of TB among herds: close relations among neighbouring herds (number and proximity of pastures) are certainly the main factors [5, 6]. However, the more recent discovery of *M. bovis* in wild animal means the potential role of wildlife involvement cannot be excluded [7]. Indeed, *M.°bovis* has been detected in deer, wild boar, badger and even fox populations in most regions where TB problems in cattle persist [8, 9].

## The recent evolution of control strategies

Eradication of TB is the ultimate objective of the French national program and its success relies on a great deal of common sense and a rational approach [10]. In order to overcome the difficulties encountered in recent years, since 2010 the revised control program objectives include:

- To move towards a risk-based strategy to reduce new infections mainly by managing factors linked to breeding practices and wildlife: a national monitoring system for wildlife has been initiated [8] and legislation on wildlife biosecurity issues have been implemented [11],
- to pursue infected herd elimination mainly by better diagnosis at herd level and at the abattoir:
- public health authorities provide training courses for slaughterhouse monitoring and authorized veterinary surgeons performing the skin test [12]
- inclusion of the interferon gamma test and PCR [13] to provide additional diagnostic sensitivity and rapid diagnostic responses [14, 15],
- to obtain the commitment of the different partners involved in the program (local authorities, veterinarians, abattoir staff, farmers) by explaining the importance of their involvement in the program for each other and for its overall success,
- to generate technical and harmonized scientific data to be used to pilot the new program.

In the framework of these objectives, the epidemiology surveillance platform in animal health (ESA Platfom) created in 2010, and at its beginning devoted to TB, contributed greatly to moving the control campaign forward. The ESA Platform (www.plateforme-esa.fr) helps to develop, adapt and promote surveillance systems by involving different stake holders and field actors in the reevaluation of the program through *ad hoc* working groups, steering committees for both cattle and wildlife surveillance programs in order to reach consensual actions and to endorse policy implementation. This platform is composed of public and private actors, farmer’s organisations, veterinary and hunter associations, research institutions, diagnostic structures, national and regional governmental actors.

One of the main prevention measures adopted consisted of reinforcing biosecurity in the farm, through guidelines drawn up by a working group under the ESA Platform, and coordinated by the French National Federation of Animal Health Defense Associations (GDS France), and the French General Directorate for Food (DGAL), under the Agriculture Ministry, organizing mandatory official training and enhancing communication as well as evaluating and following up the measures implemented.

*Mycobacterium bovis* infection was first described in free-ranging wildlife in France in 2001, with subsequent detection in hunter-harvested ungulates and badgers in areas where outbreaks of TB were also detected in cattle. Increasing concerns regarding TB in wildlife led the French General Directorate for Food (DGAL) and the main institutions involved in animal health and wildlife management, to establish a national surveillance system, known as "Sylvatub", for TB in free-ranging wildlife. The system coordinates the activities of various national and local partners. The main goal of Sylvatub is to detect and monitor *M.* *bovis* infection in wildlife through a combination of passive and active surveillance protocols adapted to the estimated risk level in each area of the country. Sylvatub is nowadays coordinated by the French Biodiversity Agency (OFB) and followed up through a steering committee constituted within the ESA platform and the active participation of all involved field actors (8). Sylvatub’s findings allowed us to realise the complexity of the situation France is facing [16] and to adapt the control program and strategies for effective disease control.

## Current epidemiological situation: identification of key elements and key constraints of the program

**Fig. 3:**
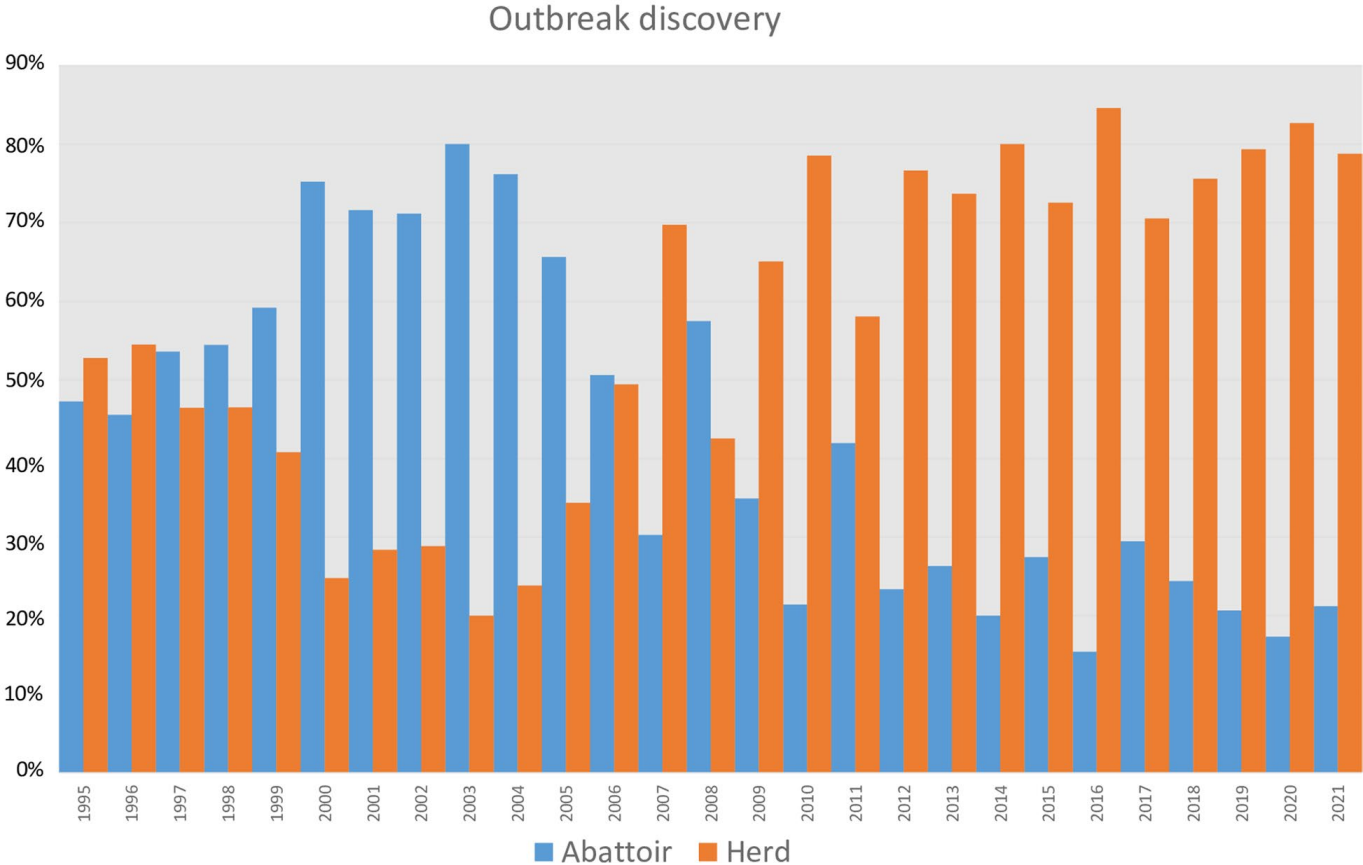
Sources of TB outbreaks from 1995 to 2021 (Source: annual report of the French General Directorate for Food (DGAL))

Since the implementation of the new adapted control measures commenced in 2010, disclosure methodologies of new outbreaks has improved: there has been a clear increase in the proportion of infected animals discovered at ante mortem testing (in the herd) in contrast to that disclosed at the abattoir. This shows that the current measures are detecting new outbreaks earlier and thus preventing further *M. bovis* transmission (Fig. 3).

**Fig. 4:**
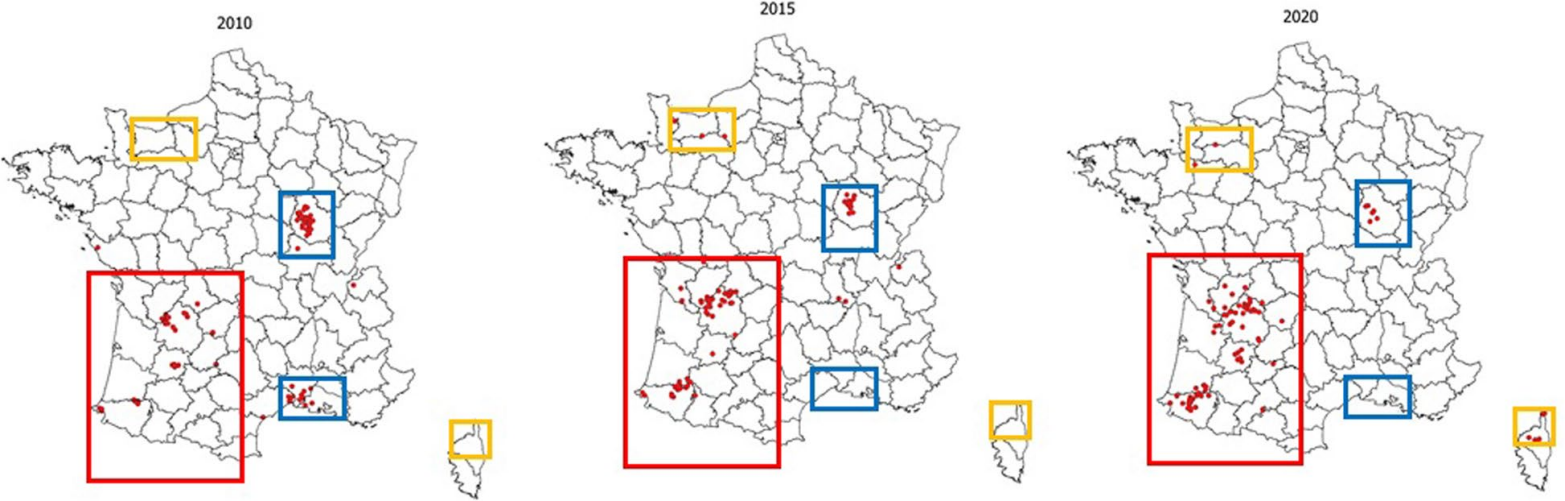
Evolution of cattle TB outbreaks in 2010, 2015 and 2020. Red dots on the maps represent cattle outbreaks. Red box: TB deterioration; yellow box: TB reemergence; blue box: TB improvement (Source: annual reports of the Direction générale de l'alimentation (DGAL))

Nonetheless, the reduction in the number of new outbreaks occurring has stalled with an equilibrium of disclosure of new outbreaks in some regions on the one hand and an improvement in the epidemiological situation with a decrease in the number of outbreaks in others (Fig. 4).

In Fig. 4, the regions in blue, Burgundy center east, and Camargue south east, are those where a clear improvement has been seen in the last ten years. This success can be attributed to the strong motivation of local field actors supported, in the longer term, by the encouraging results provided by the screening tests (good quality skin testing (3] and interferon gamma release assay (IGRA) [14, 15] and the cull strategy employed. This demonstrated that improving the information provided to veterinarians, thus increasing their awareness, was effective in improving surveillance efficacy [12]. Furthermore, it confirms the study reported by Crozet and colleagues [17] where they demonstrated that the performance of a higher proportion of comparative tests together with a more positive perception of surveillance and control programs by veterinarians were associated with better skin-test practices.

In yellow, Fig. 4 also shows those regions, such as in the north in Normandy and the Island of Corsica, where TB seems to reemerge and where improved surveillance may lead to the discovery of additional new outbreaks in future years. In these regions, provided all stakeholders show strong commitment, the situation can eventually be controlled. However, in the regions, in the southwest of France, surrounded in red in Fig. 4, where more than 80% of current outbreaks are observed, with an increase of the number of new outbreaks and an expansion of the infected zones, the situation is less favourable. In these latter areas, surveillance and management still need to be improved and better adapted according to both the epidemiological context and breeding practices.

## Eradication follow up identification of key lessons learned and next new measures

The Crozet et al study [17] also demonstrated several areas of noncompliance with regulatory recommendations regarding the skin test (bad choice of injection site, quality of injection, method of test reading, reporting of non-negative results) in several regions, mainly those less confronted with TB. Thus, instead accepting a poorly conducted test programme throughout the country, it was decided that, from the start of the prophylactic campaign 2022-2023, ante mortem testing (comparative skin test and IGRA including ESAT6-CFP10) would be concentrated on the most affected zones, reinforcing the testing regime on an annual basis. The reduction in the number of regions performing routine ante mortem testing for TB surveillance necessitates, that postmortem detection at the abattoir be as comprehensive as possible. A working group was therefore constituted, coordinated by the DGAL with ESA Platform members, to ameliorate the quality of abattoir TB surveillance. The objectives being the establishment of pertinent functional indicators to animate abattoir surveillance at a national and local level, to favour information exchange for an inherent improvement of the system, to evaluate the quality of abattoir data so as to find amelioration steps, to identify field requirements and expectations to improve training provisions and to enrich and harmonise abattoir inspection guidelines.

In terms of epidemiological investigation, *M. bovis* whole genome sequence studies were recently introduced in France. They have already provided useful data for understanding the origin of outbreaks and to improve epidemiological follow up by revealing local *M. bovis* transmission patterns in sympatric cattle and wildlife populations sharing local unique classic genotypes [18, 19]. Phylodynamics studies have also been undertaken that allow us to understand transmission patterns in the complex multi-host domestic-wild transmission cycles [20].

Availing of the valuable data from Northern Ireland badger test and vaccinate or remove (TVR) studies [21], in particular on the logistics and resources required, France has planned to undertake a similar pilot badger intervention over a 4-year period starting at the beginning of 2023, in a zone in the southwest with a serious TB problem to reinforce TB control in wildlife.

Social science can also provide guidance and advice to stakeholders so as to improve communication among field actors on prevention and control of TB and to increase acceptability of the heretofore implemented strategy of the eradication programs (G. Ciaravino, personal communication, https://innotub.eu). Some propositions to improve communication include to create more forums for sharing information and opinions, to explore common solutions proposed by different field actors, mainly farmers and the administration, to further explain testing methodologies and administrative strategies to farmers, provide better guidance for veterinarians, to strongly support farmers so that they get public acknowledgement of their important role in the society and to include the actors even more at decision taking regarding TB.

## Conclusions

Together with all the measures already undertaken and planned for the future, it is essential to sustain ongoing national research together with international collaborations in order to generate relevant scientific data to enabling national and local authorities to identify and undertake the most pertinent measures for the program and to tackle the disease in the short term.

## Data Availability

Not applicable.

## Abbreviations

ANSES: Agence nationale de sécurité sanitaire des aliments, de l’environnement et du travail
bTB: Bovine tuberculosis
TB: Animal tuberculosis
DGAL: Direction Générale de l'Alimentation
ENVA: Ecole Nationale Vétérianire d’Alfort
ESA: Epidémiologie en santé animale
GDS: Groupement de défense sanitaire
IGRA: Interferon gamma release assay
NRL: Societé nationale reference laboratory
OTF: Officialy tuberculosis free
SNGTV: Societé nationale des groupements techniques vétérinaires
TVR: Test vaccinate or remove
UAB: Universidad Autónoma de Barcelona
